# The impact of social support and emotion dysregulation on COVID-19 depressive symptoms

**DOI:** 10.3389/fpsyg.2023.1165889

**Published:** 2023-08-24

**Authors:** Deepali M. Dhruve, Jenna E. Russo, Arazais D. Oliveros

**Affiliations:** Department of Psychology, Mississippi State University, Starkville, MS, United States

**Keywords:** social support, emotion regulation, depression, pandemic, emerging adults

## Abstract

**Introduction:**

Stress resulting from the global COVID-19 pandemic has been linked to psychological consequences, such as depressive symptoms, for individuals worldwide. Outbreaks and pandemics are known to accentuate stressors or generate new ones owing to health-related worries, reduced mobility, and social activity due to quarantine, and sudden life changes. Although post-lockdown U.S. research findings suggest a greater risk of depression among 18- to 25-year-olds, familiarity with technology and virtual socializing may offer college students some protective effect, warranting research with such groups.

**Methods:**

The current study thus explored emotion dysregulation (ED) and perceived social support (PSS) as potential mechanisms for the relationship between COVID-19 stress and depressive symptoms among students at a southern university in the United States. Participants (*N* = 489) completed a cross-sectional survey assessing their current levels of COVID-19 stress, ED, PSS, and depressive symptoms.

**Results:**

Path analysis showed that PSS buffered the effect of ED on depressive symptoms. The results support the explanatory role of ED in the relationship between COVID-19 stress and depressive symptoms.

**Discussion:**

The perceived social connection may be an essential factor for psychological outcomes during periods of stress and isolation, particularly for those reporting high ED.

## Introduction

The SARS coronavirus disease that emerged in December 2019 (COVID-2019) was declared an international pandemic by the World Health Organization in March 2020 (Cucinotta and Vanelli, 2020). There is a crucial need to examine the impacts of COVID-19 on mental health to inform intervention and policy (Gruber et al., 2020). Studies have found that COVID-19 is associated with a significant increase in depressive symptoms in emerging adults (Hawes et al., 2022), with a longitudinal study by Alzueta et al. (2021) finding that the risk for clinical depression tripled among emerging adults. However, there is a need for further investigation to fully understand the underlying mechanisms of this relationship. From the literature on affective disorders and depression, difficulties in emotion regulation (e.g., Visted et al., 2018) and social support (e.g., Alsubaie et al., 2019) have been identified as factors involved in symptomatology and functioning. The current study aimed to understand how emotion regulation and perceived social support (PSS) may promote resilience and mitigate the long-term psychological impacts of stressful events.

Emotion dysregulation (ED) has been identified as a transdiagnostic process across various mental health outcomes (Beauchaine and Cicchetti, 2019). Depression is one such mental health outcome that has been attributed to biological stress system dysregulation (Vinkers et al., 2021). Prior studies have established that ED mediates the stress–psychopathology relation (Mclaughlin and Hatzenbuehler, 2009; Moriya and Takahashi, 2013). According to the stress-buffering hypothesis, PSS is another factor that may reduce adverse outcomes related to stress (Cohen and Wills, 1985). A meta-analysis of 16 studies from different countries' college students found that the prevalence of depressive symptoms was the highest among U.S. students (Chang et al., 2021), further underscoring the necessity to investigate underlying mechanisms for the relationship between COVID-19 stress and depressive symptoms. To the best of our knowledge, the current study is among the first to explore potential mechanisms for the relationship between COVID-19 and depressive symptoms in college students in the USA.

Many interacting influences are thought to serve as pathways to depression, including genetic risk, with heritability estimates ranging from 30 to 45% (Sullivan et al., 2000; McMahon, 2018). Genetic vulnerability influences neurobiological processes that shape early temperament consisting of sensitivity to harmful stimuli, disposition to feeling negative affect, and high negative emotionality, with these vulnerabilities likely interacting with environmental events to account for substantial variance (Sullivan et al., 2000). Environmental stressors serve as the catalyst or trigger for a depressive disorder to be expressed (diathesis-stress model). There is an abundance of evidence that exposure to environmental stressors, including potentially traumatic events (PTEs), is associated with increased symptoms of depression. Examples include PTEs related to natural disasters (Goldmann and Galea, 2014), terrorist events (Galea et al., 2002), and other epidemics such as Ebola (Jalloh et al., 2018), severe acute respiratory syndrome (Hawryluck et al., 2004), and most recently—the COVID-19 pandemic (Hawes et al., 2022).

Extant literature has demonstrated that ED mediates the stress–psychopathology relation (Mclaughlin and Hatzenbuehler, 2009; Moriya and Takahashi, 2013). A developmental framework for depression includes a series of neurobiology-related factors, including individual differences in temperament, information processing biases, stressful events, executive functioning, and self-regulation (Hankin, 2012). A significant amount of research supports that ED plays a role in the onset, overlap, and maintenance of depressive symptoms (Fitzgerald et al., 2019). Furthermore, experimental manipulation, resulting in improved emotion regulation, can predict subsequent improvements in symptom severity (Radkovsky et al., 2014). These findings are further supported by meta-analyses, indicating that individuals with remitted major depressive disorder (MDD) report improved emotion regulation abilities (e.g., Visted et al., 2018), emphasizing emotion regulation as a potential strategy to prevent depressive symptoms from reaching clinically significant levels. In light of the COVID-19 pandemic, more recent research has demonstrated the protective role of emotion regulation in mitigating the relationship between COVID-19-related stress and psychological symptoms ratings, including ratings of depression (Russo et al., 2022).

According to the stress-buffering hypothesis, initially proposed by Cassel (1976), social support is another factor that may reduce adverse outcomes related to stress (Cohen and Wills, 1985). A literature review by Gariepy et al. (2016) found that social support is associated with lower levels of depressive symptoms among adults. However, according to the stress-mobilizing hypothesis (Barrera, 1986), stress can also encourage individuals to seek support, warranting further research to understand the association. To date, social support has been broadly construed in two ways: perceived social support (PSS) and received social support (Eagle et al., 2019). PSS refers to an individual's subjective perception of support (material, psychological, etc.) from others and has been shown to be more strongly associated with mental health outcomes when compared to associations with received social support (Santini et al., 2015). PSS may be particularly important during periods of social distancing due to the COVID-19 pandemic. For instance, research demonstrates a positive association between PSS and individuals' self-ratings of resilience (Killgore et al., 2020). Furthermore, research on depressive symptoms during the COVID-19 pandemic found that individuals with lower PSS, compared to those endorsing high PSS, were at double the risk for elevated depressive symptoms (Grey et al., 2020). This finding is further supported by previous research showing that PSS is a significant predictor of depressive symptoms (Alsubaie et al., 2019). Additionally, previous research indicates that PSS can reduce the association between exposure to disasters, such as flood exposure and depressive symptoms (e.g., Dar et al., 2018). To the best of our knowledge, however, whether PSS could moderate (i.e., reduce the strength of) the relationship between COVID-19-related stress in particular and depressive symptoms has yet to be examined.

Prior studies have established that ED mediates the stress–psychopathology relation (Mclaughlin and Hatzenbuehler, 2009; Moriya and Takahashi, 2013) and that social support may moderate/buffer the risk for depression (Cohen and Wills, 1985). Emerging adulthood, a period of developing emotion regulation capacity, may be a susceptible period for developing depression (see Kuwabara et al., 2007). Furthermore, a recent study revealed that participants without depressive symptoms showed a greater increase in symptoms during the COVID-19 pandemic, while those with pre-existing mental health disorders showed a slight symptom increase (Pan et al., 2021). This finding suggests that the impact of the COVID-19 pandemic on depressive symptoms in individuals without pre-existing symptoms is multifaceted and requires further investigation. Thus, the current study explored potential mechanisms for the relation between COVID-19 stress and depressive symptoms in undergraduate students since college is traditionally a period of emerging adulthood. In particular, the current study proposed the following hypotheses: (1) COVID-19 stress would increase the risk for depressive symptoms, (2) ED would mediate the relationship between COVID-19 stress and depression, and (3) PSS would moderate the stress–depression association. This study aimed to help identify modifiable risk factors involved in mental health outcomes during pandemics by exploring these mechanisms.

## Method

### Participants

Kline (2015) suggested that a sample size of 200 or more is appropriate for path analysis. The current sample included 489 participants (341 women, 148 men, and no others) ranging in age from 18 to 58 years (*M* = 20.18, *SD* = 4.35) attending a large university in the southern region of the United States. Although the participant pool sampled for the current study included 16 participants older than the traditional threshold for emerging adulthood, all participants (mainly in their 20s) were undergraduate college students and are thus referred to as emerging adults (Arnett, 2007). Much of the sample reported their race as Caucasian/white (77.9%), followed by African American/Black (18.6%), Asian (3.9%), American Indian/Alaska Native (2.0%), and Native Hawaiian/Pacific Islander (0.4%), or others (1.0%). See Table 1 for the sociodemographic characteristics of the participants.

**Table 1:** Sociodemographic characteristics of participants.

**Characteristics**	***n***	**%**
**Sex**		
Female	341	69.7%
Male	148	30.3%
**Academic year**		
Freshman	262	53.6%
Sophomore	82	16.8%
Junior	68	13.9%
Senior	75	15.3%
**Race**		
Caucasian/white	381	77.9%
African American/Black	91	18.6%
Asian/Pacific Islander	19	3.9%
American Indian/Alaska Native	10	2.0%
Native Hawaiian/Pacific Islander	2	0.4%
Other	5	1.0%
**Ethnicity**		
Hispanic/Latino	21	4.3%
Non-Hispanic/Latino	466	95.7%
**Parents yearly income**		
Less than $10,000	15	3.1%
$10,000–$19,999	15	3.1%
$20,000–$29,999	16	3.3%
$30,000–$39,999	37	7.6%
$40,000–$49,999	23	4.7%
$50,000–$59,999	38	7.8%
$60,000–$69,999	44	9.0%
More than $70,000	34	7.0%

*N* = 489. Participants were on average 20.18 years old, ranging from 18 to 58 years.

### Procedure

Upon approval by the university's Institutional Review Board, participants were recruited through an undergraduate research pool at a southern university in the United States between November 2020 and April 2021. Participants completed all procedures remotely using their computers due to COVID-19 restrictions. Thus, some participants may have been attending classes in person at school, whereas others may have been at home attending classes virtually. Participants accessed the survey using a link to Qualtrics. Qualtrics has been used in several COVID-19-related studies (Czeisler et al., 2021; Johnson, 2021; McFadden et al., 2022). First, informed consent was obtained *via* Qualtrics. Next, the participants provided demographic data, including gender, age, and race/ethnicity. The participants then completed the battery of measures. Each measure was presented in a randomized order to account for order effects. After the participants completed the survey, they were provided information about on-campus psychological services. The participants received course credit as compensation for their participation.

### Measures

The online questionnaire included the COVID Stress Scale (CSS; Taylor et al., 2020), the Difficulties with Emotion Regulation Scale (DERS; Gratz and Roemer, 2004), the Child and Adolescent Social Support Scale-College version (CASSS-C; Malecki et al., 2000), Beck Depression Inventory-II (BDI-II; Beck et al., 1996), and a demographic questionnaire.

The CSS (Taylor et al., 2020) is a 36-item self-report measure assessing COVID-19-related stress and anxiety symptoms. It includes subscales for COVID-19 danger and contamination fears, COVID-19 fears about economic consequences, COVID-19 xenophobia, COVID-19 compulsive checking and reassurance seeking, and COVID-19 traumatic stress symptoms. Items are scored on a 5-point scale: 0 (*not at all*), 1 (*slightly*), 2 (*moderately*), 3 (*very*), and 4 (*extremely*). The CSS has demonstrated good psychometrics, with the internal consistency of the scales ranging from 0.86 to 0.95, plus good convergent and discriminant validity (Taylor et al., 2020). The current study used the overall CSS score to measure participants' COVID-19 stress. The CSS demonstrated good internal consistency (α = 0.87) in our sample.

The DERS (Gratz and Roemer, 2004) is a 36-item scale that measures the following facets of ED: non-acceptance, goal-directed behavior, impulsivity, emotional awareness, strategy, and clarity. Participants responded on a 5-point scale (1 = almost never; 5 = almost always). Scores ranged from 0 to 180, with higher scores indicating greater emotion dysregulation. Based on physiological and neural indicators of emotion regulation, the DERS total score represents a reliable global index of overall ED (Gratz et al., 2006; John and Eng, 2014). The DERS demonstrates high internal consistency with Cronbach's alpha ranging from 0.77 to 0.93 (Gratz and Roemer, 2004; Dhruve and Oliveros, 2022). Conversely, discriminant validity evidence for the DERS subscales is limited (John and Eng, 2014). Thus, the DERS total score was used in the present study to measure ED.

The CASSS-C (Malecki et al., 2000) is a modified version of the CASSS; it has been utilized in a prior COVID-19 study (Balkundi and Fredrick, 2023). The CASSS-C is a 60-item self-report measure that assesses the perceived frequency and importance of support from four sources: family, other adults (e.g., professors and coaches), peers, and close friends. The current study utilized frequency items to measure participants' overall PSS. Frequency items are scored on a 6-point scale (1 = never and 6 = always). Frequency scores ranged from 60 to 360, with higher scores representing a greater perceived frequency of social support. The CASSS-C demonstrated good internal consistency (α = 0.87) in our sample.

The BDI-II is a 21-item self-report measure (Beck et al., 1996) that assesses depressive symptoms. The BDI-II has been used in several COVID-19-related studies (Bashir et al., 2020; Leão et al., 2023; Zabel et al., 2023). Items are scored on a Likert scale from 0 to 4, with higher scores specifying more depressive symptoms. The severity of depressive symptoms is represented as follows: 0–13 is the minimal range, 14–19 is the mild range, 20–28 is the moderate range, and 29–63 is the severe range. The BDI-II demonstrated excellent internal consistency (α = 0.94) in our sample.

### Statistical analysis

Categorical variables were described using frequencies and percentages. Continuous variables were described using means and standard deviations. Path analyses were performed to evaluate the relationship between COVID-19 stress, emotion regulation, social support, and depression. Standardized regression coefficients were calculated, and R2 measures for each exogenous variable were estimated. Goodness-of-fit criteria were calculated to assess model fit with comparative fit index (CFI) ≥ 0.90, Tucker–Lewis index (TLI) ≥ 0.90, and root mean square error of approximation (RMSEA) < 0.10 indicating acceptable model fit. Analysis was performed using IBM SPSS AMOS (version 27.0). Missingness occurred at less than 5% and was handled by imputation. Data were checked for normality and multicollinearity and were found to be within normal limits. All skew, kurtosis, and variance inflation factor statistics were < 1.2.

## Results

Table 2 provides an overall characterization of the sample. Participants reported low levels of COVID-19 stress although there was considerable variability in responses (*M* = 26.63, *SD* = 23.73). On average, participants endorsed moderate levels of PSS (*M* = 257.59, *SD* = 56.29), but ratings ranged from 60 to 360, indicating that the full range of this measure is reflected in our current sample. ED scores ranged from 36 to 163, which reflects most of the possible range of scores on this measure (DERS maximum = 180). Despite there being no clinical cutoffs for the DERS, 53% of participants reported DERS scores in the second percentile, indicating that at least 20 out of 36 items were endorsed as causing difficulties most of the time. All variables correlated as expected.

**Table 2:** Descriptive statistics and bivariate correlations.

**Variable**	***M***	***SD***	**1**.	**2**.	**3**.	**4**.
1. COVID-19 stress	26.63	23.73	1	–	–	–
2. Perceived social support	257.59	56.29	−0.13^*^	1	–	–
3. Emotion dysregulation	88.48	25.48	0.31^*^	−0.29^*^	1	–
4. Depressive symptoms	12.76	11.25	0.25^*^	−0.38^*^	0.58^*^	1

*n* = 489.

^*^p < 0.01.

As shown in Table 2, correlational analyses revealed that COVID-19 stress was positively associated with depressive symptoms (*r* = 0.25, *p* < 0.001) and ED (*r* = 0.31, *p* < 0.001). As expected, PSS was negatively correlated with COVID-19 stress (*r* = −0.19, *p* < 0.001), depressive symptoms (*r* = −0.38, *p* < 0.001), and ED (*r* = −0.32, *p* < 0.001). Depressive symptoms were positively correlated with ED (*r* = 0.58, *p* < 0.001).

**Figure 1:**
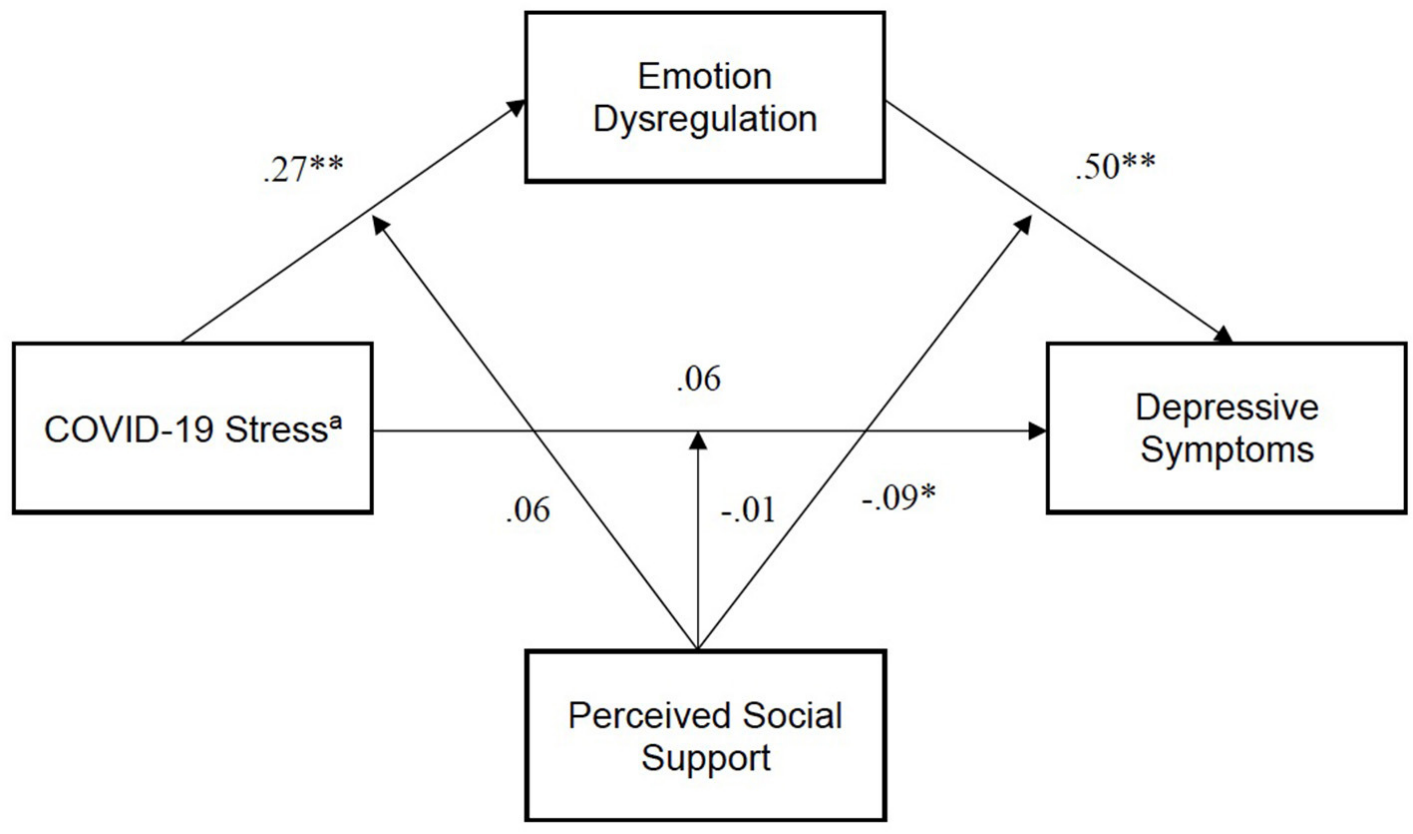
Path analysis model. χ^2^(1) = 3.70, *p* = 0.54, CFI = 0.99, SRMR= 0.02. Errors omitted for clarity. Exogenous variables freely correlated. aIndirect effect of COVID-19 stress on depressive symptoms = 0.14, 95% CIs (0.09, 0.18). *p < 0.05, **p < 0.01.

**Figure 2:**
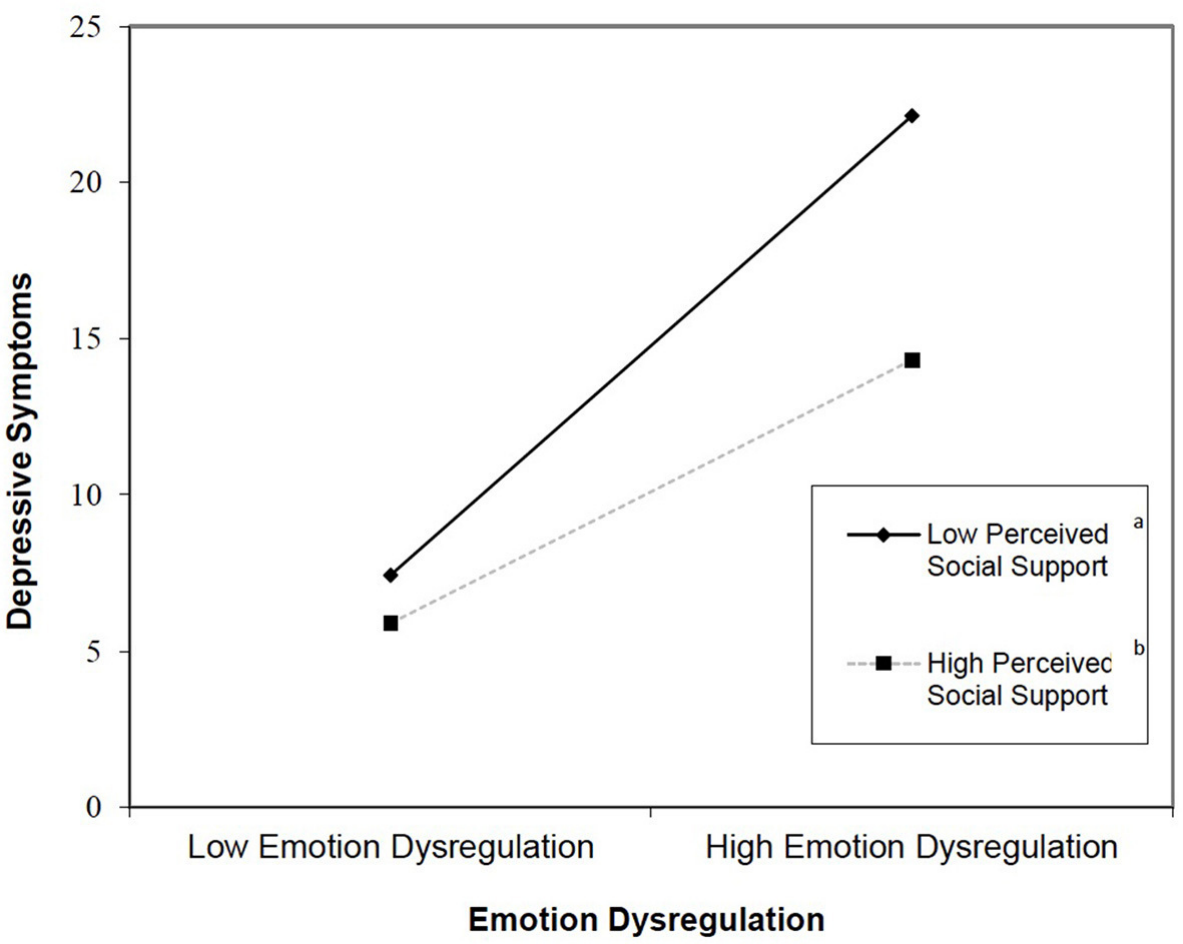
Plot of interaction effect of emotion dysregulation and social support on depressive symptoms. ^a^*m* =0.29. ^*b*^*m* = 0.17.

All hypotheses were tested using the structural model in Figure 1. Hypothesis 1, COVID-19 stress would increase the risk for depressive symptoms and be partially supported as COVID-19 stress had an indirect effect on depressive symptoms through ED [*b* = 0.14, 95% BCa (0.09, 0.18), *p* < 0.001]. Although COVID-19 stress did not have a significant direct effect on depressive symptoms, the modern method for mediation analysis does not require evidence of a simple association between the predictor and outcome variables to estimate and test hypotheses about indirect effects (Hayes and Rockwood, 2017). This result fully supported hypothesis 2, that ED would mediate the association between COVID-19 stress and depression. Contrary to hypothesis 3, that PSS would moderate the stress–depression association, PSS did not moderate the relationship between COVID-19 stress and ED. However, PSS significantly moderated the interaction between ED and depressive symptoms (*b* = −0.09, *p* < 0.05), resulting in moderated mediation in an unexpected way. As shown in Figure 2, lower PSS increased the risk of depressive symptoms for those with higher ED, even for those with lower ED, however, lower PSS was associated with higher levels of depressive symptoms.

## Discussion

The current study investigated ED and PSS as potential mechanisms in the relationship between COVID-19 stress and depressive symptoms. The present findings highlight the role of ED as a mechanism between COVID-19 stress and depressive symptoms, supported by previous research that describes ED as a transdiagnostic risk factor for adverse psychological outcomes (e.g., Beauchaine and Cicchetti, 2019). In concordance with the stress-buffering hypothesis (Cassel, 1976), in the current study, the effect of ED on depressive symptoms varied as a result of PSS. Specifically, participants with high ED and low PSS were at greater risk of reporting depressive symptoms than those with high PSS.

Although correlational analyses demonstrated that PSS negatively correlated with COVID-19 stress and ED, regression analysis suggested that PSS did not moderate the relation between COVID-19 stress and ED. Thus, the effect of COVID-19 stress on ED did not vary across differing levels of PSS. Stated differently, PSS did not protect individuals from the dysregulating impact of COVID-19 stress. Since PSS does not significantly alter the relationship between COVID-19 stress and ED, this suggests that other factors, such as coping strategies or personality traits, may be more important in determining how individuals respond to COVID-19 stress and its impact on ED (Al-Omiri et al., 2021; Liu et al., 2021; Chankasingh et al., 2022). In contrast, the risk of depressive symptoms was lower for those with more PSS, even when endorsing higher levels of ED. Individuals with high ED may perceive social support as less supportive, even if they have equal social support as someone with low ED. The stress-mobilizing hypothesis (Barrera, 1986) suggests that stress can encourage individuals to seek support, while the stress-buffering hypothesis (Cassel, 1976) explains that social support can reduce negative outcomes associated with stress. This means that the association between COVID-19 stress and PSS could be positive for some (i.e., more stress leading to more support seeking) and negative for others (i.e., more support seeking to lead to less stress).

### Clinical implications

Given the known increase in loneliness among students and young adults during the COVID-19 lockdown and the social distancing required by public health guidelines (Bu et al., 2020), the protective role seen here for PSS, decreasing the risk for depressive symptoms, highlights the importance of increasing perceived social connection during periods of stress. Even when physical health requires social distancing and quarantining, the mental health needs of individuals merit health communication/campaigns that balance physical safety with psychological functioning. The current findings indicate that perceived social connection may be a critical consideration for those endorsing higher ED.

It should be noted that PSS does not significantly lessen the pathway from COVID-19 stress to ED. This suggests an enduring relationship between stress and ED. Given this finding, mental health messaging should also raise awareness of emotion regulation. Whereas most people talk about depression, fewer may know to pay attention to fluctuations in emotion regulation, and the framing of health information is known to impact people's attitudes and behaviors, including public stigma and help-seeking (Devendorf et al., 2020). Increasing awareness about emotion regulation skills and their role in mental health may help mitigate the long-term psychological impact of pandemics on college students.

Incorporating virtual group skills sessions to cultivate emotion regulation skills among college students may benefit universities. Research in the field has established the utility of group therapy in enhancing emotion regulation abilities (Joormann and Stanton, 2016; Wimmer et al., 2019; Carroll et al., 2023). Furthermore, providing virtual group sessions may engender a sense of enhanced PSS that can help offset the deleterious impact of quarantine measures during a pandemic (Marmarosh et al., 2020). Virtual group sessions may also help foster emotion regulation skills and social connection among other populations, including those who have experienced disasters. For example, virtual group sessions may benefit those living in rural areas with limited access to services.

### Limitations

These associations should be considered within the context of their limitations. Considering its correlational, cross-sectional design, the current study cannot explain the directionality of the relations between the variables or infer causation. However, it offers insight into the mechanisms underlying pandemic-related stress and depressive symptoms. As the current study utilized a convenience sample, the results may not generalize across all college campuses or adults. Additionally, we cannot conclude that COVID-19 stress caused perceived social isolation nor that it was the sole reason for higher depressive symptoms. However, the interplay of these variables appears to explain a significant amount of variance, which can inform future research and intervention. Additionally, being from a rural region in the United States, the responses from participants in this sample may differ from those from urban areas since rural counties are associated with higher case and mortality rates (Huang et al., 2021).

### Future research directions

Future studies should select a sample characterized by diversity in race and ethnicity, geographic regions, socioeconomic status, and educational status to determine if such factors impact the relationship between stress, ED, PSS, and depressive symptoms. The question of whether the combination of contextual and intra-individual factors, e.g., may lead to differing appraisals of social support, or differing forms of emotion regulation, remains unanswered. Furthermore, the role of race and ethnicity may be critical factors to investigate (Dyer, 2020; Lopez et al., 2020) since cultural display rules (Malatesta and Haviland, 1982) may inform emotion regulation processes, and the cultural meaning of social support has been demonstrated to affect one's willingness to rely on social networks during stressful times (Taylor et al., 2004; Bareket-Bojmel et al., 2021). Finally, future studies should consider utilizing longitudinal designs to clarify the directionality of the relations between the variables or to infer causation.

## Conclusion

The current study sheds light on the potential mechanisms involved in the relationship between COVID-19 stress and depressive symptoms among college students. The findings suggest that ED plays a significant role in this relationship, and PSS can buffer the effect of ED on depressive symptoms. These results highlight the importance of increasing perceived social connection during periods of stress, as it may decrease the risk of depressive symptoms, especially for those with high ED. The current findings have important implications for mental health interventions during pandemics and periods of stress. Health communication campaigns should balance physical safety with psychological functioning and emphasize the importance of both social connection and emotion regulation skills. By doing so, we may be able to mitigate the long-term psychological impact of pandemics on college students and promote better mental health outcomes.

## Data availability statement

The datasets presented in this article are not readily available because the data are available from the authors upon reasonable request. Requests to access the datasets should be directed at: AO, aoliveros@psychology.msstate.edu.

## Ethics statement

The studies involving human participants were reviewed and approved by Mississippi State University Institutional Review Board. The patients/participants provided their written informed consent to participate in this study.

## Author contributions

DD: conceptualization and methodology. DD, JR, and AO: manuscript drafting, review, and editing. AO and JR: data analysis. All authors contributed to the article and approved the submitted version.
